# Emergent Diagnosis of a Flail Mitral Leaflet With Bedside Echocardiography

**DOI:** 10.7759/cureus.9374

**Published:** 2020-07-24

**Authors:** Patrick Hsu, Caroline Shepherd, Keyon Shokraneh, Gabriel Cabrera, Eric J Kalivoda

**Affiliations:** 1 Emergency Medicine, Hospital Corporation of America West Florida Graduate Medical Education Consortium/Brandon Regional Hospital, University of South Florida Morsani College of Medicine, Brandon, USA

**Keywords:** flail mitral leaflet, mitral regurgitation, point-of-care ultrasound, focused cardiac ultrasound, echocardiography, emergency department

## Abstract

Flail mitral leaflet (FML) with associated acute pulmonary edema and acute decompensated heart failure is an emergent condition requiring prompt recognition by the emergency physician (EP). Focused cardiac ultrasound (FOCUS) and lung ultrasound (LUS) have a vital role in the evaluation of FML in the emergency department. This case report describes the identification of a FML with EP-performed bedside echocardiography.

## Introduction

Focused cardiac ultrasound (FOCUS) and lung ultrasound (LUS) are critical point-of-care tools for the rapid evaluation and management of acute decompensated heart failure (ADHF) in the emergency department (ED) [1-4]. The sonographic features that support the diagnosis of ADHF include the presence of a diffuse B-line lung profile and left ventricular (LV) systolic and/or diastolic dysfunction. The echocardiographic assessment of valvular pathology in the context of acute pulmonary edema (APE) can be challenging in the emergent setting; it is also important to note that valvular assessment is considered an advanced skill for emergency physicians (EPs) [5]. Significant valvular dysfunction, specifically mitral regurgitation (MR), is likely present in a substantial cohort of ED patients with APE [6]. Mitral valve (MV) dysfunction should be routinely considered as a contributing etiology of ADHF by the EP [7].

Myxomatous degenerative disease of the MV apparatus can ultimately lead to notable complications, such as severe MR and flail mitral leaflet (FML) [8]. The emergent identification of FML is often difficult given that the history, physical examination, and chest radiography lack diagnostic sensitivity. FML can be initially misdiagnosed in 60% of cases when relying on clinical variables alone. Echocardiography is imperative for the early recognition and timely management of ADHF due to FML with severe MR. A previous case report described the utility of bedside echocardiography for identifying acute MV insufficiency (ultimately diagnosed by formal echocardiogram as FML secondary to ruptured chordae) in the clinical context of new-onset congestive heart failure [9]. To the best of our knowledge, there are only two other previous reports that have described EP-performed FOCUS in the emergent diagnosis of FML [10,11]. This case report highlights the pivotal role that an EP-performed FOCUS and LUS had in initiating timely ED management for a patient with ADHF and a suspected FML.

## Case presentation

A 60-year-old male patient with reported past medical history of mitral valve prolapse (MVP) presented to the ED with the chief complaint of right flank pain. He described a severe, constant, right flank pain radiating to his right lower abdominal quadrant with associated nausea and vomiting for approximately one hour prior to arrival. He denied fevers, chills, back pain, testicular pain or swelling, urinary frequency or urgency, hematuria, dysuria, diarrhea, constipation, or bloody stools. The patient also endorsed worsening dyspnea on exertion and orthopnea over the previous two weeks with associated intermittent, mild, sharp, non-radiating substernal chest pain. He denied lightheadedness, palpitations, syncope, lower extremity swelling, cough, or hemoptysis.

Initial vitals were temperature 36.8°F, blood pressure 109/70 mmHg, heart rate 76 beats per minute with a regular rhythm, respiratory rate 18 breaths per minute, and oxygen saturation of 95% on room air. On initial physical examination, the patient was in no acute distress and non-toxic appearing, mild right flank and right lower quadrant abdominal tenderness were present without rebound, guarding, or peritoneal signs. A holosystolic murmur was appreciated best at the apex. The patient was in no respiratory distress and lungs were clear to auscultation. Electrocardiogram (ECG) revealed normal sinus rhythm without acute ischemic changes. Portable chest radiography demonstrated slight interstitial prominence. CT of the abdomen and pelvis, laboratory studies, urinalysis, an intravenous fluid (IVF) bolus, antiemetic, and pain medications were ordered as his initial presentation was suggestive of an intra-abdominal process. Additionally, CT pulmonary angiography (CTPA) was ordered for clinical suspicion of an acute intrathoracic process.

**Figure 1:**
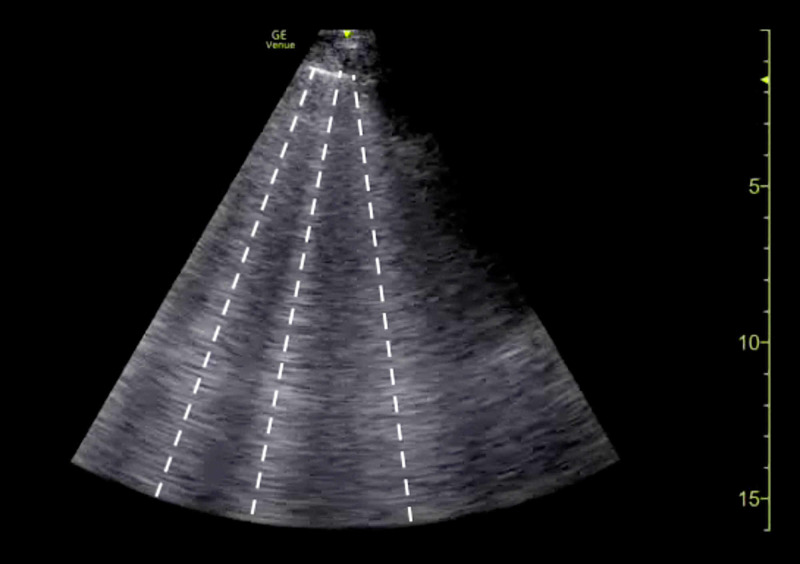
Point-of-care lung ultrasound demonstrating a B-line profile (white lines).

**Figure 2:**
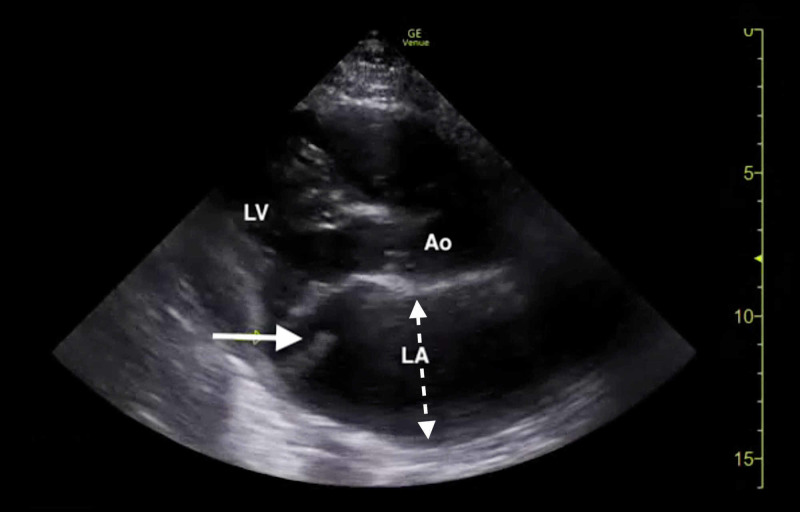
Bedside transthoracic echocardiogram (parasternal long-axis window) demonstrating a flail posterior mitral leaflet (solid arrow) and left atrial dilation (dotted arrow). Ao, aortic root; LA, left atrium; LV, left ventricle.

**Figure 3:**
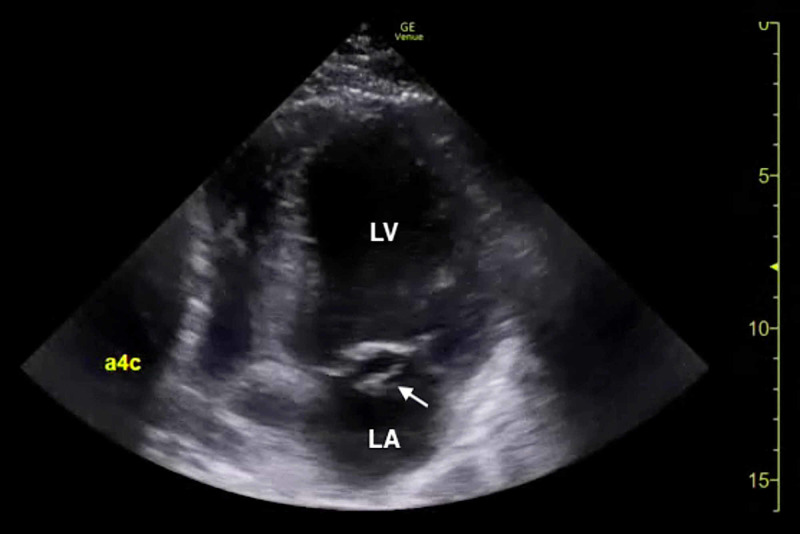
Bedside transthoracic echocardiogram (apical window) demonstrating a flail posterior mitral leaflet (arrow). LA, left atrium; LV, left ventricle.

The clinical history was also concerning for an undiagnosed cardiomyopathy, particularly given the heart murmur appreciated on examination and his stated history of MVP for which the patient had never sought outpatient cardiology follow-up. Bedside LUS and FOCUS were then performed by emergency medicine resident physicians. LUS was notable for the presence of a diffuse B-line profile consistent with pulmonary edema (Figure 1). FOCUS was unremarkable for evidence of pericardial effusion, signs of right heart strain, or decreased LV contractility. However, FOCUS revealed a dilated left atrium (LA) and a suspected posterior FML, with the posterior MV leaflet seen protruding into the LA (Figures 2, 3; Videos 1, 2).

**Video 1:** Bedside transthoracic echocardiogram (parasternal long-axis window) demonstrating a flail posterior mitral leaflet.

**Video 2:** Bedside transthoracic echocardiogram (apical window) demonstrating a flail posterior mitral leaflet.

**Figure 4:**
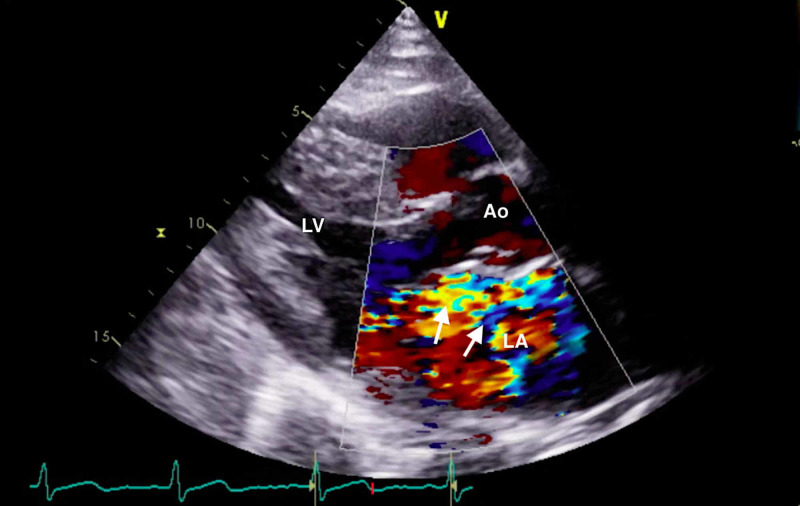
Comprehensive transthoracic echocardiogram (parasternal long-axis window) with color Doppler demonstrating flail motion of the posterior mitral leaflet and a severe eccentric mitral regurgitant jet (arrows). Ao, aortic root; LA, left atrium; LV, left ventricle.

**Figure 5:**
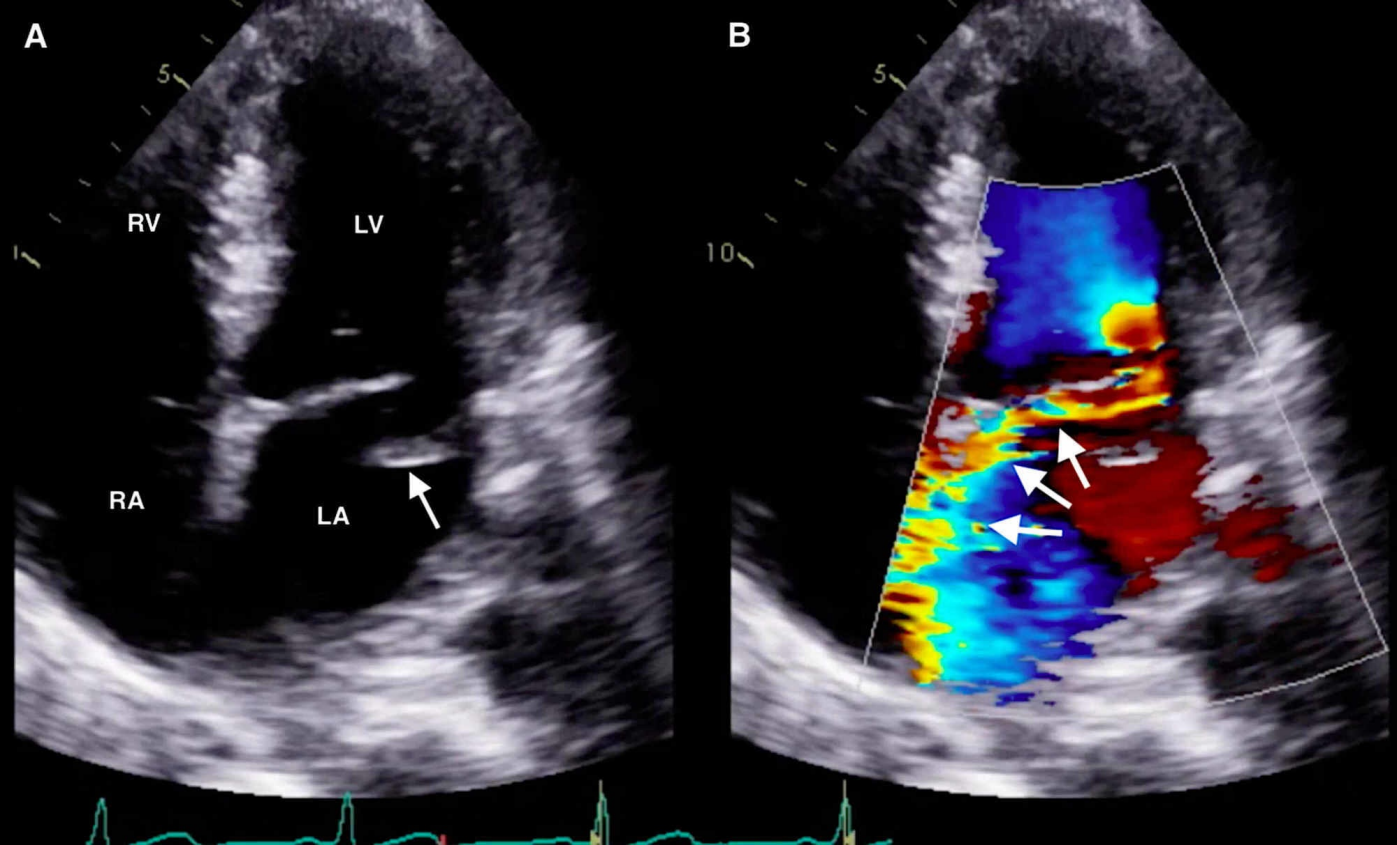
Comprehensive transthoracic echocardiogram (apical four-chamber window) demonstrating (A) flail motion of the posterior mitral leaflet and (B) severe eccentric mitral regurgitant jet (arrows). RA, right atrium; RV, right ventricle; LA, left atrium; LV, left ventricle.

The constellation of FOCUS findings immediately prompted a formal comprehensive transthoracic echocardiogram (TTE) and emergent cardiology and cardiothoracic surgery consultations. TTE confirmed preserved LV systolic function, LA dilation, and flail motion of the posterior MV leaflet with severe eccentric MR (Figures 4, 5; Videos 3-5).

**Video 3:** Comprehensive transthoracic echocardiogram (parasternal long-axis window) with color Doppler demonstrating flail motion of the posterior mitral leaflet and severe mitral regurgitation.

**Video 4:** Comprehensive transthoracic echocardiogram (apical four-chamber window) with and without color Doppler demonstrating flail motion of the posterior mitral leaflet and severe mitral regurgitation.

**Video 5:** Comprehensive transthoracic echocardiogram (apical four-chamber window with expanded zoom) demonstrating flail motion of the posterior mitral leaflet.

Laboratory analysis was notable for white blood cell count of 13.9 (4.0-10.5 × 10^3^/µL), creatinine 1.58 (0.7-1.3 mg/dL), troponin I 0.184 (0-0.045 ng/mL), and N-terminal pro B-type natriuretic peptide (NT-proBNP) of 1,075 (0-450 pg/mL). Urinalysis revealed hematuria without evidence for urinary infection. CT of the abdomen and pelvis confirmed a 2 x 4 mm calculus at the right ureterovesical junction with mild to moderate right-sided hydronephrosis. CTPA was negative for pulmonary embolism or aortic pathology,, however, was significant for cardiomegaly, pulmonary vascular congestion, and trace bilateral pleural effusions consistent with congestive heart failure.

The patient’s clinical status deteriorated rapidly during the latter ED course from presumed APE secondary to FML. On repeat examination, he had developed mild to moderate respiratory distress and his vital signs were blood pressure 103/63 mmHg, heart rate 68 beats per minute, respiratory rate 21 breaths per minute, and oxygen saturation of 85% on 4 L nasal cannula. Due to his acute change in clinical condition with the ED diagnosis of APE secondary to FML, non-invasive positive pressure ventilation and intravenous diuresis were subsequently initiated. 

**Figure 6:**
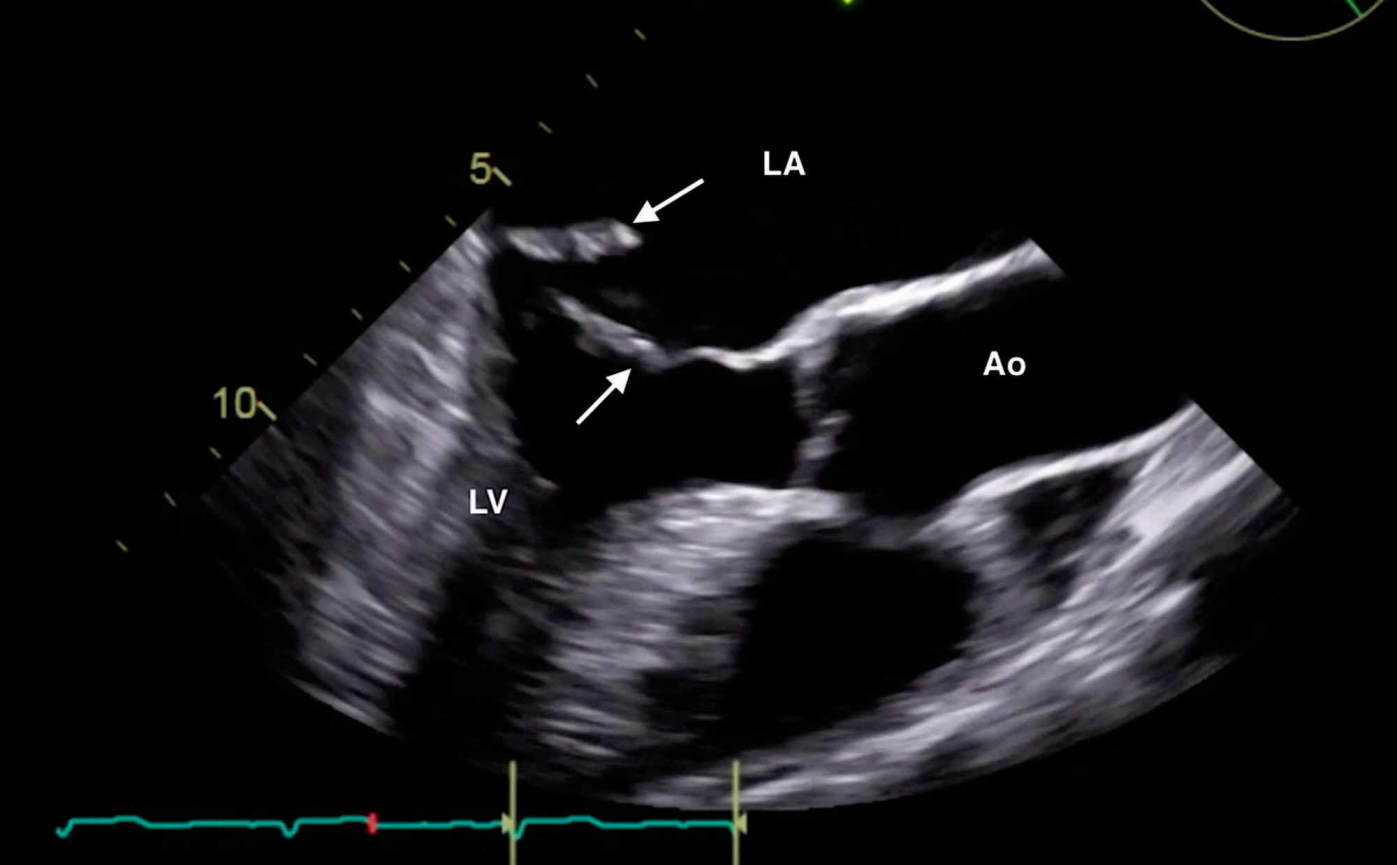
Transesophageal echocardiogram demonstrating normal position of the anterior mitral leaflet (rightward-pointing arrow) and flail motion of the posterior mitral leaflet (leftward-pointing arrow). Ao, aortic root; LV, left ventricle; LA, left atrium.

The patient was admitted to the cardiovascular intensive care unit (CVICU) for transesophageal echocardiogram (TEE), cardiac catheterization, and cardiothoracic surgery consultation. TEE demonstrated preserved LV systolic function, severe flail motion of the posterior mitral leaflet, and severe MR (Figure 6; Video 6). Cardiac catheterization was performed and demonstrated angiographically normal coronary arteries. Cardiothoracic surgery performed MV repair with resection of the flail P2 segment and MV annuloplasty. The patient was ultimately discharged on hospital day 9 after a stable and uneventful postoperative course.

**Video 6:** Transesophageal echocardiogram demonstrating flail motion of the posterior mitral leaflet.

## Discussion

The major etiologies of acute MR include non-ischemic causes such as spontaneous ruptured chordae tendineae, infective endocarditis, and myxomatous leaflet degeneration and myocardial ischemia with associated papillary muscle rupture [12,13]. MVP is caused by myxomatous MV degeneration and has an estimated prevalence of 2%-3% [13,14]. MVP is the most common cause of severe non-ischemic MR in the United States, yet with only a small subset of patients developing major complications. There are two described forms of MVP, “classic” prolapse with myxomatous thickening of the mitral leaflets and “non-classic” prolapse with a more anatomically normal MV with lesser degrees of leaflet thickening; individuals with “non-classic” MVP have a better prognosis and less complications [13,15]. MVP may also be categorized by unileaflet and bileaflet subsets; interestingly, patients with unileaflet MVP have a higher incidence of flail leaflets than those with bileaflet MVP [16]. Our case patient likely had chronic “non-classic” unileaflet MVP which had degenerated to a posterior FML. It is notable that LA dilation was identified on bedside echocardiogram, suggesting that he had undiagnosed chronic MR. In contrast, patients with acute MR will have normal LA size. Consistent with our patient’s ultimate diagnosis, patients with FML will predominantly have severe MR with an eccentric mosaic regurgitant jet, usually with involvement of the P2 segment [17].

Bedside ultrasonography is an essential tool in the ED evaluation of APE [1-4]. EPs should be mindful that patients presenting in acute respiratory distress with APE may infrequently have an underlying FML with severe MR [8,12]. Our patient's acute worsening of clinical status due to APE was possibly triggered by the IVF bolus he received early in the ED course; however, resuscitative management was cautiously pursued given the EP knowledge of likely early-onset pulmonary edema based upon initial LUS examination. It is common for an underlying FML to be misdiagnosed as ADHF on initial presentation and consequently reduce the urgency for emergent echocardiogram, therefore leading to significant and detrimental delays in FML diagnosis [8]. This report highlights the critical importance of prompt EP recognition of FML with bedside echocardiography, as confirmatory comprehensive echocardiogram and specialist consultations were expedited in the ED. A delayed FML diagnosis is often associated with increased morbidity and mortality, and early surgical MV repair reduces long-term mortality and cardiovascular complications [8,18-20]. Advanced EP-performed echocardiography has an integral and evolving role in emergent cardiac conditions previously not considered under the domain of the non-cardiologist [2,5]. 

## Conclusions

Early recognition of APE due to FML with EP-performed echocardiography is critical to facilitating the appropriate ED management of this life-threatening condition. Timely diagnosis of FML with subsequent early surgical intervention can lead to improved mortality outcomes. Future ED-based studies are warranted to determine the clinical impact of EPs performing goal-directed valvular assessment with bedside echocardiography.
